# *Escherichia coli *genes affecting recipient ability in plasmid conjugation: Are there any?

**DOI:** 10.1186/1471-2164-10-71

**Published:** 2009-02-09

**Authors:** Daniel Pérez-Mendoza, Fernando de la Cruz

**Affiliations:** 1 Departamento de Biología Molecular (Universidad de Cantabria) and Instituto de Biomedicina y Biotecnología de Cantabria (CSIC-UC-IDICAN), C/Herrera Oria s/n, 39011 Santander, Spain; 2 Department of Biochemistry, University of Cambridge, Cambridge, CB2 1QW, UK

## Abstract

**Background:**

How does the recipient cell contribute to bacterial conjugation? To answer this question we systematically analyzed the individual contribution of each *Escherichia coli *gene in matings using plasmid R388 as a conjugative plasmid. We used an automated conjugation assay and two sets of *E. coli *mutant collections: the Keio collection (3,908 *E. coli *single-gene deletion mutants) and a collection of 20,000 random mini-Tn*10*::Km insertion mutants in *E. coli *strain DH5α. The combined use of both collections assured that we screened > 99% of the *E. coli *non-essential genes in our survey.

**Results:**

Results indicate that no non-essential recipient *E. coli *genes exist that play an essential role in conjugation. Mutations in the lipopolysaccharide (LPS) synthesis pathway had a modest effect on R388 plasmid transfer (6 – 32% of wild type). The same mutations showed a drastic inhibition effect on F-plasmid transfer, but only in liquid matings, suggesting that previously isolated conjugation-defective mutants do in fact impair mating pair formation in liquid mating, but not conjugative DNA processing or transport *per se*.

**Conclusion:**

We conclude from our genome-wide screen that recipient bacterial cells cannot avoid being used as recipients in bacterial conjugation. This is relevant as an indication of the problems in curbing the dissemination of antibiotic resistance and suggests that conjugation acts as a pure drilling machine, with little regard to the constitution of the recipient cell.

## Background

Plasmids are self-replicating mobile genetic elements. They are separate from the chromosome and contain a specific subset of genes from the bacterial genetic pool [1,2]. Many plasmids conjugate between different bacteria, especially related ones, leading to intra- and inter-specific dissemination of plasmid-specific genes, for instance, antibiotic resistance genes. As a result, virtually identical plasmids are isolated repetitively in different bacterial species [3,4]. We and others consider that inhibition of plasmid dissemination by inhibiting conjugation might be a useful strategy to enhance or complement the efficacy of antibiotics and curb the isolation of antibiotic resistant bacterial pathogens [5-7]. With this objective in mind, we proposed to learn which genes in the recipient bacteria are needed for the production of transconjugants.

In 1968, Curtiss *et al. *[8] suggested already that conjugation required the active participation of both mating partners. They demonstrated an association between energy metabolism in the F^- ^parent and the rate of chromosome transfer in Hfr × F^- ^matings. During the 70s, the use of different strategies facilitated the identification of recipient functions implicated in conjugation. For example, lethal zygosis (the death of F^- ^cells in an Hfr × F^- ^cross), a phenomenon initially described by Clowes [9], allowed the possibility of selecting for mutants in conjugation recipient ability [10]. Resistance to lethal zygosis was accompanied by alterations in membrane functions (transport and accumulation of galactosides; [9]). It was initially thought that the transfer of a large amount of DNA from an Hfr donor was responsible for recipient killing. However, F^- ^recipients were also killed when transfer was blocked by adding nalidixic acid to mating mixtures (nalidixic acid inhibits the formation of transconjugants without appreciable impact on mating aggregate formation). This fact suggested that extensive damage in the recipient membrane was the primary cause of lethal zygosis [11]. Other strategies involved the use of mutants producing bacteriophage-resistance or colicin-tolerance, since both were found to be resistant to conjugation [12,13]. Skurray *et al. *[14] coined the term "Con^- ^mutants". A Con^- ^mutant was defective as a conjugation recipient with either F' or Hfr donor strains. They were distinguished from recombination-deficient mutants (e.g., *recA*), which were unable to inherit DNA from Hfr donors but not from F' donors. The Con^- ^mutants identified by Skurray [14] lacked OmpA, a major outer membrane protein, and resulted in 0.1 to 1% of the parental conjugation frequency. In addition, certain lipopolysaccharide (LPS) mutants of *S. typhimurium *and *E. coli *were also described as Con^- ^mutants [15]. They differed from Skurray's Con^- ^mutants both in the sugar composition of the membrane as well as in an altered permeability for certain antibiotics. Subsequently, it was demonstrated that the phosphate diester bridges in the LPS backbone as well as some membrane proteins are important in forming a cell surface structure resistant to the penetration of several antibiotics [16]. In summary, these experiments provided evidence for the involvement of specific recipient cell surface components (LPS and OmpA) in conjugation. Unfortunately, most cell envelope mutants were obtained by treatment of recipients with random mutagens [17,18] so the mutated sites could not be exactly determined. More recently, other studies focused on the identification of plasmid gene products that interact with the recipient surface. It was shown that TraN and TraG are involved in recipient cell recognition as well as in entry exclusion of F-like plasmids [19,20]. In the IncI1 plasmid R64, recipient recognition is mediated by expression of seven plasmid-encoded PilV adhesins. The PilV adhesins are thought to be located at the tips of the thin R64 pili in the donor cell. The different sequences for the C-terminal segments of PilV adhesins are produced by shufflon DNA rearrangement and they determine the recipient specificity in liquid matings of plasmid R64 through the recognition of LPS on the surface of recipient cells. For instance, PilVA adhesin recognizes the GlcNAc (β1–3) Glc moiety of *E. coli *R1 type LPS [21,22].

Here, we present a high-throughput screening (HTS) strategy to systematically evaluate the implication of each individual *E. coli *gene in bacterial conjugation. We used an automated *lux*-monitored conjugation assay to screen a collection of 20,000 random Tn-insertion *E. coli *mutants as well as a collection of 3,908 deletion mutants in each individual *E. coli *gene (the Keio collection [23]). Our strategy allowed us to cover > 99% of the non-essential *E. coli *genome and allowed us to gain a general understanding of the implication of *E. coli *functions in recipients of bacterial conjugation.

## Results

### Implementation of a HTS *lux*-monitored conjugation assay to detect conjugation mutants in recipient cells

The principle of the HTS conjugation assay relies on the production of visible light by pSU2007::Tn*lux*, a derivative of plasmid R388. pSU2007::Tn*lux *contains a *lux *operon under the control of a *lac *promoter. Expression of *lux *in conjugative donor cells is repressed by the *lac *repressor LacI, encoded on a co-resident and non-mobilizable multicopy plasmid (pUC18::*lacI*^*q*^). Upon conjugation, pSU2007::Tn*lux*, but not pUC18::*lacI*^*q*^, is transferred to recipient cells and thus light is produced. There is a direct relationship between light emission and frequency of conjugation [6]. Ninety-four independent cultures of *E. coli *strain DH5α were tested as recipients to examine the reproducibility of the assay. The donor strain was UCDPM1 [CSH53 (pSU2007::Tn*lux *+ pUC18::*lacI*^*q*^)]. The control assay was validated by the use of two different negative controls: the donor strain alone (UCDPM1), and DH5α expressing the entry exclusion protein (Eex) of R388 [Eex_R388; DH5α (pSU5024); [6]]. Eex inhibits transfer of a plasmid to a recipient cell harbouring the same element [24], and thus it is equivalent to a conjugation recipient mutant.

**Figure 1:**
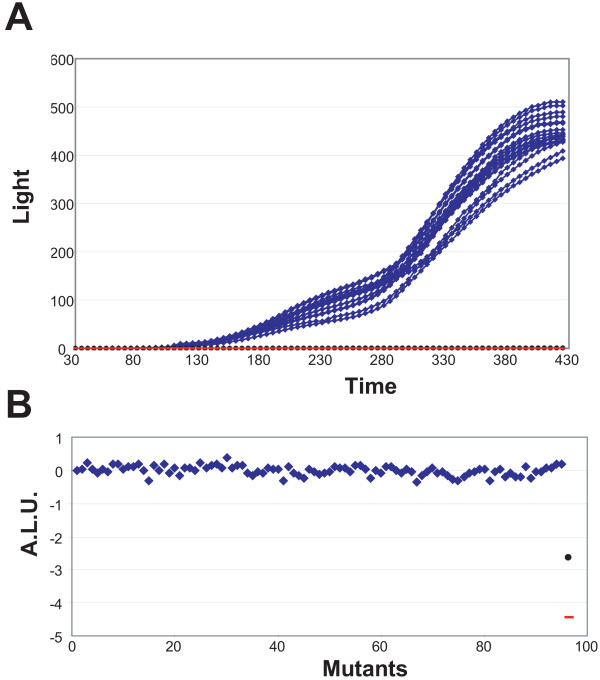
**Analysis of conjugation by the light emitted in the HTS assay**. (A) The time-course of light emission in 94 individual DH5α wt conjugation mixtures (blue diamonds), donor alone (UCDPM1; red dash) and DH5α expressing Eex_R388 [DH5α (pSU5024); black circle] is shown. (B) Representation of the results in (A) as arbitrary light units [ALU = log_10 _(Light-x/Light-wt)]. In Fig. 1A light emission of only 20 of the 94 DH5α colonies are represented in order to help in visualization.

The kinetics of plasmid transmission was analysed by following light production versus time (Fig. 1A). Maximum light values were obtained after 400 min conjugation at 37°C (Fig. 1A). The median of the arbitrary light units (ALU; see Methods) produced by the 94 DH5α colonies was -0.0097 with values ranging between +0.3702 and -0.3732. When DH5α (pSU5024) was used as a recipient, light production was more than 500-fold lower than with DH5α (ALU = -2.71; Fig. 1B), underscoring the role of the Eex protein in avoiding redundant conjugation. Furthermore, light production of UCDPM1 was 10,000 times lower (ALU = -4.13; Fig 1B) confirming the tight control of light production in donor cells. The small dispersion of light values (σ_ALU _= 0.1435) in addition to the low quantity of light produced by the negative controls confirm that the automated conjugation assay was suitable for HTS of recipient conjugation mutants.

### Screening of *E. coli *chromosomal genes involved in bacterial conjugation using Keio collection

**Figure 2:**
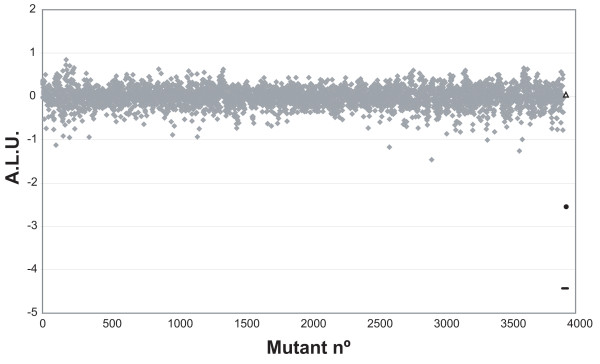
**ALU values of Keio collection mutants**. ALU values [ALU = log_10 _(Light-x/Light-wt)] of the HTS conjugation assay when applied to the 3,908 Keio collection mutants (grey diamonds), BW25113 wt strain (black triangle), donor without recipient (UCDPM1; black dash) and BW2511 expressing Eex_R388 [BW25113 (pSU5024); black circle)]

**Figure 3:**
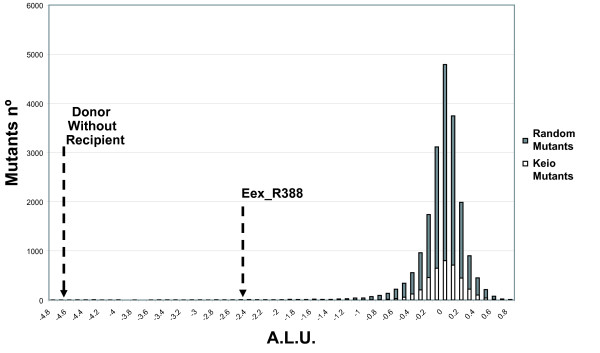
**Distribution of mutants ALU values**. Distribution of ALU values [ALU = log_10 _(Light-x/Light-wt)] of the HTS conjugation assay when applied to the 3,908 Keio collection mutants (white bars) or to 20,000 miniTn*10::kan *insertion mutants (grey bars).

The 'Keio collection' comprises a set of single-gene in-frame deletion mutants of most non-essential genes of *E. coli *and therefore provides a useful resource for genome-wide testing of mutational effects [23]. In order to test the Keio collection as recipients in conjugation, the donor strain UCDPM1 was conjugated to each of the 3,908 Keio collection mutants in 96-well microplates using a Biomek2000^® ^robot. Light production was measured and ALU values calculated as described in Methods. A representation of ALU values for the 3,908 individual clones is shown in Fig. 2. The wt recipient strain (BW25113), the donor strain (UCDPM1) and the recipient expressing Eex_R388 [BW25113 (pSU5024)] were included as controls. The median ALU produced by the Keio mutants was -0.010, with values ranging between +0.846 and -1.459 (σ = 0.22). Their distribution is shown in Fig. 3. None of the 3,908 mutants tested showed a decrease in light production comparable to Eex_R388, indicating that none of the mutations abolished plasmid R388 conjugation (Fig. 2). However, several mutants showed reduced light production. The 38 mutants that showed ALU = -0.643 (lowest 1% of total) were re-assayed together with the wt strain (8 separately grown colonies) and the appropriate negative controls [UCDPM1 and BW25113 (pSU5024)]. ALU values for the mutants were now calculated considering Light-wt as the average light value of the 8 wt conjugation mixtures. Among the re-assayed mutants only five showed ALU ≤ -1 (equivalent to at least a 10-fold drop in light production; Table 1). These mutants were then checked in a standard plate conjugation assay and their transfer frequencies calculated. The *rfaC *mutation resulted in a 5-fold reduction, the *uvrD *mutation resulted in a 2.5-fold reduction and the *ihfB*, *rimM *and *ybeX *mutations resulted in 2-fold or less reduction in conjugation frequency (Table 1). This result distinguishes between effects in conjugation proper and other effects that could limit the HTS recorded frequencies, such as effects on *lux *expression or the bioluminescence reaction, and thus confirm that none of the Keio mutants results in a dramatic drop in the conjugation frequency.

**Table 1:** Conjugation frequencies of a selected subset of the Keio collection mutants

**Recipient strain**	**Gene mutated and relevant features**	**ALU**^a^	**Relative transfer frequency in standard assay (% of wt)**^b^
No recipient		-4.37	<10^-6^
BW25113 (pSU5024)		-2.55	<10^-6^
K1:F4	*ihfB*: Integration host factor, beta subunit	-1.44	98
K83:B10	*rfaC*: Inner core LPS biosynthesis	-1.28	22
K29:D12	*uvrD*: Subunit × of helicase II (DNA repair enzyme)	-1.25	41
K69:C4	*rimM*: 16S rRNA processing protein	-1.03	48
K25:F8	*ybeX*: Putative integral membrane protein; magnesium and cobalt transporter	-1.02	55

^a ^Arbitrary light units produced by the given mutant in the HTS conjugation assay (see text for details).

^b ^Results of a standard 1 h plate conjugation assay (see Methods). Results are shown relative to the parental BW25113 strain. Transfer rates are the average of at least three independent experiments.

### Screening for *E. coli *genes involved in bacterial conjugation by using Tn insertion mutants

Since screening the Keio collection did not uncover any *E. coli *genes with a major role in R388 conjugation, random mutagenesis with mini-Tn*10*::Km [25] was carried out in order to generate an additional set of *E. coli *mutants for HTS. In contrast to Keio mutants, transposon insertion can generate mutations that inactivate more than one gene (e.g., a full operon) and, more importantly, mutations that affect genes at different levels (e.g., mutants that modulate gene expression by insertions in promoter regions [26], or mutants with partial gene activity by insertions near the C-terminus of a gene).

**Figure 4:**
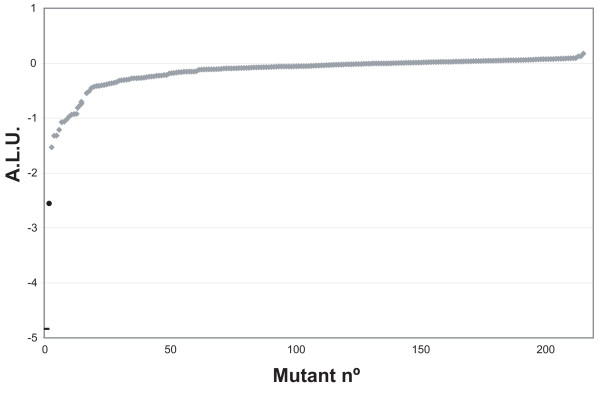
**Result of the HTS conjugation assay when applied to a selected subset of the 20,000 mini-Tn*10*::Km insertion mutants**. The HTS conjugation assay was run on a collection of 20,000 mini-Tn*10*::Km insertion mutants as described in Methods. A subset of 237 mutants, which gave ALU ≤ -1 in the first assay were selected for a second assay. The figure shows the ALU values [ALU = log_10 _(Light-x/Light-wt)] of these 237 mutants plotted in increased order (grey diamonds), together with donor without recipient (UCDPM1; black dash) and DH5α expressing Eex_R388 [DH5α (pSU5024); black circle].

**Figure 5:**
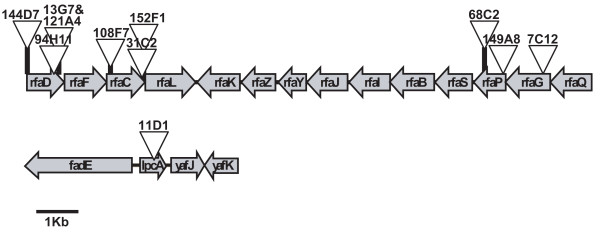
**Insertions located in LPS biosynthesis genes**. Genetic localization of 11 mini-Tn*10*::Km insertions in *E. coli *genes involved in three LPS biosynthesis operons. Transposon insertion positions are shown by triangles that identify mutant numbers.

A mutant library was constructed by random insertion of mini-Tn*10*::Km in the *E. coli *chromosome. A strategy to map transposon insertions was designed based on inverse PCR (see Methods). Twenty thousand DH5α Km^r ^mini-Tn*10*::Km insertion mutants were grown individually and conjugated with donor strain UCDPM1 in 96-well plates as described in Methods. ALU values were calculated for each mutant. The median ALU produced by the mutants was -0.046 (σ = 0.34). Their distribution is shown in Fig. 3. Among the 20,000 mutants, 237 showed ALU ≤ -1 (representing a 10-fold drop in light production). Twenty-two mutants showed only residual growth in liquid media and were eliminated from the screening (data not shown). The remaining 215 mutants were re-assayed together with the appropriate controls. ALU values for these mutants were re-calculated considering Light-wt as the average light value of the 8 wt conjugation mixtures. Only 15 mutants showed ALU ≤ -0.6 (Fig. 4). Genomic DNA was isolated from these strains and the transposon insertion point was identified (Table 2). The transfer rate of plasmid R388 to these mutants were also calculated and compared with the wt strain (Table 2). All but one of the 15 mutants showed a significant reduction in the transfer rate of R388 plasmid when used as recipients. Interestingly, in 11 of these 14 mutants the insertion was located in genes involved in LPS biosynthesis. The 11 insertions in LPS genes were located at 10 different positions and interrupted one of 5 genes (*rfaD*, *rfaC*, *rfaP*, *rfaG*, and *lpcA*) that mapped to two regions of the *E. coli *chromosome (Fig. 5). However, since transposon insertions might be polar, expression of more than one gene within LPS operons could have been affected by each insertion. As shown in Fig. 5, the insertions in LPS genes could be classified according to their location in 3 different groups: (i) Insertions 144D7, 13G7, 121A4, 94H11, 108F7, 152F1 and 31C2, located either in *rfaD *or *rfaC*, showed the highest impact in the R388 transfer (ranging between 16- and 4-fold lower than wt; Table 2). (ii) Insertion 11D1 in *lpcA *gene (also known as *gmhA*) decreased R388 transfer by 7-fold (Table 2). (iii) Insertions 68C2, 149A8 and 7C12 interrupting *rfaP *or *rfaG *decreases R388 transfer between 3- and 5-fold (Table 2). In addition, all 11 mutants showed a mucoid colony phenotype, as described previously for other LPS mutants [27], and increased susceptibility to nalidixic acid (data not shown), another reported feature of bacteria defective in LPS [16]. The remaining 3 insertions targeted non LPS-related genes *crp*, *gppA*, and *nhaA*, all with modest reductions in R388 transfer (Table 2).

**Table 2:** Conjugation frequencies of a subset of mini-Tn*10*::Km transposon mutants

**Recipient strain**	**Gene mutated and relevant features**	**A.L.U**.^a^	**Relative transfer rate in standard assay (% of wt)**^b^
No recipient		-4.84	<10^-6^
DH5α (pSU5024)		-2.57	<10^-6^
108F7	*rfaC*: Lipopolysaccharide biosynthesis	-1.53	6
129B10	*crp*: DNA-binding transcriptional dual regulator	-1.32	59
183G6	*yejM*: Hypothetical phosphatase/sulphatase	-1.32	96
94H11	*rfaD*: Lipopolysaccharide biosynthesis	-1.21	13
152F1	*rfaC*: Lipopolysaccharide biosynthesis	-1.07	20
130F12	*gppA*: Guanosine pentaphosphatase/exopolyphosphatase	-1.07	16
13G7	*rfaD*: Lipopolysaccharide biosynthesis	-1.03	23
121A4	*rfaD*: Lipopolysaccharide biosynthesis	-1.02	22
144D7	*rfaD*: Lipopolysaccharide biosynthesis	-0.97	6
11D1	*lpcA*: Lipopolysaccharide biosynthesis	-0.94	15
31C2	*rfaC*: Lipopolysaccharide biosynthesis	-0.92	26
149A8	*rfaP*: Lipopolysaccharide biosynthesis	-0.79	21
68C12	*rfaP*: Lipopolysaccharide biosynthesis	-0.76	32
110B7	*nhaA*: integral membrane Na+/H^+ ^antiporter	-0.74	41
7C12	*rfaG*: Lipopolysaccharide biosynthesis	-0.60	19

^a ^Arbitrary light units produced by the given mutant in the HTS conjugation assay.

^b ^Normalized to the wild type (DH5α) and expressed as a percentage. Transfer rates are the average of at least three independent experiments.

### Using the *lux*-monitored conjugation assay to evaluate the transfer rate of F plasmid to the previously identified LPS mutants

**Figure 6:**
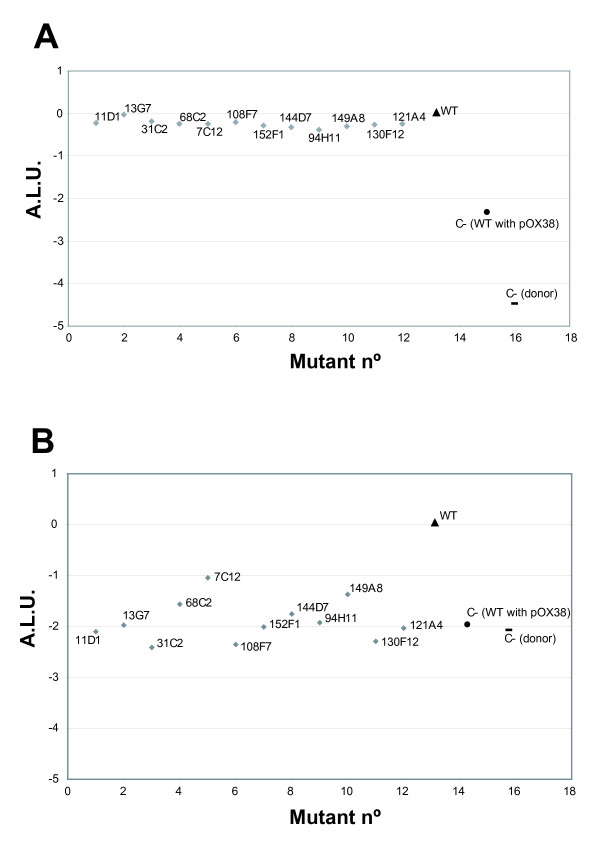
**ALU values of the F-*lux*-monitored conjugation assay**. ALU values [ALU = log_10 _(Light-x/Light-wt)] of the F-*lux*-monitored conjugation assay when applied to the LPS mutants (grey diamonds) in solid (A) or liquid (B) mating conditions. DH5α wt strain (black triangle), donor without recipient (black dash) and DH5α [(pOX38); black circle].

The two mutant sets used in this study point out LPS genes as the main non-essential recipient functions implicated in R388 conjugation recipient ability. They also show that, under surface mating conditions, defective LPS in recipient cells results in only minor effects when compared to conjugation of F-like plasmids in liquid media [18]. To evaluate the recipient ability of our LPS mutants, along with the *gppA *mutant (130F12), in F plasmid conjugation, a *lux *derivative of pOX38 was constructed (See Methods). A donor strain harbouring the F-*lux *derivative [DH5α (pOX38::*lux *+ pUC18:: *lacI*^*q*^)] was conjugated with each of the 11 LPS mutants both in solid and liquid matings. The appropriate controls were included [donor without recipient and DH5α (pOX38)] and the resulting ALU values represented in Fig. 6. Under surface mating conditions, the 11 LPS mutants showed similar ALU values with respect to a wt recipient (Fig. 6A) indicating that recipient ability was unaffected. Under liquid mating conditions, however, the 11 mutants showed ALU values between -1 and -2.5 (Fig. 6B) and therefore a significant decrease in recipient ability, to the level of the negative controls used in the experiment. In addition, the 46 Keio mutants which showed the lowest ALU values in the HTS assay with R388 were also tested with the donor strain harbouring an F-*lux *derivative in solid and liquid mating conditions. Under solid mating conditions, the tested subset of Keio mutants showed ALU values comparable to the wt strain BW25113 (ALU = ± 0.5). In perfect agreement with the results above, only the mutant K83:B10 (*rfaC*) showed an important decrement in ALU values in comparison to the wild type in liquid mating conditions (ALU of K83:B10 = -1.87). Again, this value was similar to the negative controls of the experiment [ALU of donor without recipient = -2.16 and ALU of BW25113 (pOX38) = -2.11].

## Discussion

In this study we set out to determine what *E. coli *genes, if any, are required by the recipient bacteria in plasmid conjugation. Our screening assay used plasmid R388 as a test plasmid. Since it conjugates only on solid surfaces, it represents an interesting alternative to F-like plasmids, which are able to conjugate in liquid media. Previous papers reported significant differences between liquid and surface mating [10,18,28] with the effects of cell envelope mutations reduced or eliminated in surface mating. Thus a fresh analysis using HTS methods was warranted. All characterized F plasmid Con^- ^mutants were LPS or OmpA mutants (See ref. [18]). This fact was interpreted by Frost as evidence of a receptor for either the pilus or TraG/N products that mediate aggregate formation [20]. Alternatively, Taylor proposed that Con^- ^mutants were affected in the general constitution (charge) of the membrane [29]. It must be remembered that all previous attempts to identify Con^- ^mutants used enrichment procedures, such as lethal zygosis or resistance to infection by bacteriophages, in order to select for potential mutants. These procedures may bias the range of potential Con^- ^mutants to those with membrane alterations, leaving out genes involved in other functions (e.g. DNA processing reactions). In this respect, our work incorporated two substantial improvements over previous analyses. First, we used an exhaustive HTS conjugation assay and two independent sets of *E. coli *mutants that, combined, cover 99% of non-essential *E. coli *genes. Second, we used plasmid R388, a surface mater, as a test plasmid. Surface mating is genetically simpler (requiring 15 genes instead of 35) and thus concentrates on the core of the mating apparatus.

Due to our assay set up, some mutations might affect other processes besides conjugation *per se*. For example, the *ihfB *mutant showed the lowest value (ALU = -1.44) among the Keio mutants. However, a standard conjugation assay indicated that R388 plasmid transferred to an *ihfB *recipient at a similar rate to the wt (Table 1). Since it showed decreased light production in the HTS assay but a normal transfer rate in the standard conjugation assay, we assume it was affected in the process of light production. Two mutants, *crp *and *rimM*, showed a statistically significant reduction in R388 transfer in the standard conjugation assay (Tables 1 and 2), but since these genes affect a number of bacterial processes [30] we consider their effect on conjugation as scarcely informative. Among the genes regulated by CRP there are many membrane transport proteins [31], so a *crp E. coli *mutant should have membrane alterations among other negative pleiotropic effects. A mutation of *rimM*, encoding a 16S rRNA processing protein, caused a growth delay both in liquid and solid media (data not shown). Similarly to a *crp *mutant, a *rimM *mutant should be affected in numerous bacterial processes including bacterial viability, and therefore a direct implication in conjugation could not be established.

The remaining mutants showed a decrease in light production as well as a reduction of R388 transfer in standard conjugation assays, thus confirming a role in conjugation. The *uvrD *mutant showed a transfer rate of less than 50% with respect to the wt strain. The *uvrD *gene encodes DNA helicase II, which plays roles in nucleotide excision repair, mismatch repair, homologous recombination and DNA replication [32], including replication of rolling circle plasmids [33]. *uvrD *turned out to be very interesting candidate for further studies since a mutation in this gene blocks the rolling-circle replication of different plasmids after nicking [33]. The modest effect of *uvrD *mutation on R388 plasmid transfer could be a consequence of gene redundancy (a likely candidate is *rep *helicase [33]). However, most of the Con^- ^mutants we obtained were located in genes related to the recipient cell surface structure, as in the previous reports mentioned above. Mutations in two genes encoding membrane proteins NhaA and YbeX generated a slight reduction in R388 transfer (Table 1 and 2). The *nhaA *gene encodes an integral membrane Na^+^/H^+ ^antiporter [34]. YbeX is a putative integral membrane protein predicted to be involved in the transport of magnesium and cobalt ions [35].

Among the Keio mutants, *rfaC *showed the lowest R388 transfer rate in standard conjugation assays (Table 1). The *rfaC *gene encodes a heptosyltransferase I implicated in LPS inner core biosynthesis [36]. Mutations in *rfaC *produce a core-defective LPS in *E. coli *with increased permeability to a number of hydrophilic and hydrophobic agents (e.g. antibiotics; [37]), showing a mucoid colony phenotype, as described previously for other LPS mutants [27]. Interestingly, 12 additional conjugation mutants from the mini-Tn*10*::Km insertion library were identified as presenting a mucoid phenotype as well. Furthermore, the mucoid mutants showed increased susceptibility to nalidixic acid (they were unable to grow in LB agar supplemented with 20 μg/ml of the drug in contrast to the wt). Indeed, 11 of these mutants contain insertions in LPS biosynthesis genes (*rfaD*, *rfaC*, *rfaP*, *rfaG*, and *lpcA*; Fig. 5). The *rfaD *and *rfaC *genes are involved in the attachment of heptose I to 3-deoxy-D-*manno*-oct-2-ulosonic acid (KDO) in the first steps of the LPS inner core biosynthesis [37]. The *lpcA *gene (also known as *gmhA*) encodes a sedoheptulose 7-phosphate isomerase used also in the first step of the LPS inner core biosynthesis [38]. Finally, *rfaP *and *rfaG *encode functions involved in more distal assembly steps of the inner core or in the outer core of LPS biosynthesis. In principle, one would expect that the results from the two screenings carried out in this study will yield similar results. However, only a mutant in one LPS biosynthetic gene (*rfaC*) was indentified among the Keio collection. ALU values obtained from different Keio LPS biosynthetic mutants during the HTS are represented in Table 3. Although with ALU values below the threshold selected for this screening (ALU ≤ -0,643; lowest 1% of total), 12 Keio LPS mutants showed negative ALU values suggesting a defect in recipient ability. In agreement with the results obtained with the random insertion strategy, mutations in *rfaD*, *rfaF*, *rfaC *and *lpcA *genes showed the lowest ALU values. A similar trend was also observed with other mutations identified in non-LPS-related genes (e.g. *nhaA*; Keio ALU values = -0.13). The quantitative differences in ALU values obtained with each strategy could be a consequence of polar effects of mini-Tn*10*::Km insertions or due to the fact that two different *E. coli *strains were used.

**Table 3:** ALU values of Keio collection mutants in LPS biosynthesis

**Keio Mutant**	**Gene**	**ALU**^a^
K83:B10	*rfaC*	-1.28
K45:D5	*rfaF*	-0.57
K45:C5	*rfaD*	-0.39
K49:A9	*lpcA*	-0.32
K83:C10	*rfaZ*	-0.31
K45:C6	*rfaG*	-0.22
K45:D6	*rfaQ*	-0.12
K83:D10	*rfaB*	-0.12
K45:H5	*rfaI*	-0.09
K45:B6	*rfaP*	-0.09
K45:F5	*rfaY*	-0.06
K45:G5	*rfaJ*	-0.05
K45:E5	*rfaL*	0.03
K45:A6	*rfaS*	0.04

^a^Arbitrary light units produced by the given mutant in the HTS conjugation assay.

The twelfth and last mutant, 130F12, presented an insertion interrupting the coding sequence of *gppA*, generating a similar reduction in R388 transfer as the LPS mutants (Table 2). The *gppA *gene encodes a guanosine pentaphosphatase/exopolyphosphatase which is implicated in the hydrolysis of the inorganic polyphosphate chains in bacteria. Although little is known about this enzyme, the ubiquity and dynamic features of polyphosphate suggest a variety of important roles in bacteria. Its mucoid phenotype and increased susceptibility to nalidixic acid led us to think that 130F12 could be synthesizing an altered LPS.

In summary, our genome-wide screen using two independent sets of mutants did not uncover any mutations resulting in a substantial reduction of plasmid R388 conjugation. The only significant hits, the LPS mutants, resulted in transfer frequencies ranging between 16- and 4-fold lower than wt (Table 2), a minor effect. Nevertheless, using the *lux*-monitored conjugation assay with an F-*lux *plasmid, the same set of LPS mutants showed strong conjugation inhibition (Fig. 6B) suggesting a severe defect in the recipient ability in liquid mating conditions. However, recipient ability was restored when the conjugation experiments were carried out in surface mating conditions (Fig. 6A). Similar results were previously reported [10,18,28] and suggest that LPS mutants affect docking between donor and recipient in liquid medium, and not DNA processing or transport.

## Conclusion

In principle, we would expect to find two classes of recipient mutations affecting conjugation. The first class of mutants would affect entry into the recipient, by lack of a suitable receptor, or entry site, or energy for the transport process. These kinds of mutants have been found to affect phage infection and DNA transformation (see, for instance, [39,40]). The fact that we did not find mutants severely affected in these early stages suggests that conjugation does not require an active involvement of the recipient in the transport process. This idea was proposed in our "shoot and pump" conjugation model [41]. The present results reinforce our notion that the sheer push force imparted by the type four secretion system (T4SS) on the pilot protein and on the ensuing DNA is sufficient for the transport machinery to act as a syringe or a drilling machine, and inject the pilot protein and the trailing DNA into the recipient cell. From a certain point of view, we can say that bacterial recipient cells cannot avoid conjugation.

The second class of mutation we expected were mutations affecting the reforming of a replicative plasmid in the recipient cell. This process involves at least recircularization of the transferred DNA strand and synthesis of the lagging-strand. Recircularization of the transferred DNA is probably accomplished by the transported relaxase as we discussed previously [42]. Lagging-strand synthesis can be effected by a number of alternative mechanisms, as shown in the analysis of replication of ssDNA phages and rolling circle replicating plasmids [43,44]. Of these, only RNA polymerase is an essential enzyme. Genes involved in other pathways, such as *dnaG*, *priA*, *priB *and *priC *were tested as part of the Keio collection and showed no effect on conjugation. Thus, either RNA polymerase synthesizes the primer for lagging-strand synthesis as occurs for rolling circle plasmid replication [45], or the process has to be directed by a plasmid enzyme. A plasmid-encoded DNA primase exists in some conjugation systems and, interestingly, is transported to the recipient cell as well as being essential for conjugation in some heterologous matings [46]. Since plasmid R388 contains no DNA primase, it probably relies on the activity of the host RNA polymerase (which thus could be responsible, at least in part, of the host range of a conjugative system). Other gene products possibly involved in conjugative DNA processing within the recipient cells are *ssb*, DNA polymerases, DNA helicases, topoisomerases and gyrases, among others. To the extent that they were present in the tested mutants of the Keio collection, they are not required for conjugation.

What are the consequences of the fact that recipient cells contribute so little to conjugation? First, bacterial cells that want to avoid being used as recipients have to devise *ad hoc *mechanisms to inhibit conjugation. This function seems to be essential in plasmid physiology itself, and plasmids thus invented entry exclusion [24]. In fact, as shown in this work, Eex_R388 inhibits conjugation more than 500-fold, much more that the best recipient mutation found. Restriction endonucleases are an obvious alternative, which also have been shown to be powerful inhibitors of conjugation [47]. Second, there are interesting consequences for biotechnology and synthetic biology. There is now a rush to engineer minimal bacterial cells, which will be used as simpler reactors in the new biotechnological industry [48]. There is the potential doubt of whether these cells will be amenable to genetic manipulation, perhaps because they lose genes important for recipient ability in conjugation. Our data suggest that this will not be so, at least in the case of conjugation, and minimal cells will be as good recipients as their parental strains.

## Methods

### Bacterial strains and plasmids

*Escherichia coli *strains DH5α [F^- ^*supE44 lacU169 (Ô80lacZΔM15) hsdR17 recA1 endA1 gyrA96 thi-1 relA1*] [49] and BW25113 [*rrnB3 *Δ*lacZ4787 hsdR514 *Δ(*araBAD*)*567 *Δ(*rhaBAD)568 rph-1*] [23] were used as recipient strains in conjugation experiments. A derivative of strain CSH53 [*ara D(lac-pro) strA thi (Ô80ΔlacI)*] harbouring plasmid pSU2007::*Tnlux *and pUC18::*lacI*^*q *^(hereafter named UCDPM1) was used as donor [6]. Strains containing plasmid pSU5024 [6], which overproduces Eex_R388, were used as controls of poor recipient ability. Plasmid pLOF-Km [25], that contains mini-Tn*10*::Km, was used to generate random mutants in *E. coli *by direct electroporation of strain DH5α. The F-lux derivative (pOX38::*lux*) was constructed by cloning the 6 kb *NotI *fragment containing the *lux *operon, previously excised from pSU2007::*Tnlux*, into the unique *NotI *site of pOX38. When appropriate, antibiotics were added at the following concentrations: ampicillin sodium salt (Ap; 100 μg/ml), kanamycin sulphate (Km; 25 μg/ml) and nalidixic acid (Nx; 20 μg/ml).

### Bacterial electroporation

*E. coli *electroporation to generate mini-Tn*10*::Km insertions was carried out in an electro cell manipulator apparatus (BioRad). Electrocompetent cells were prepared according to the instructions of the manufacturer and stored at -80°C. For electroporation, cells were thawed on ice, mixed with pLOFKm DNA (0.3–0.5 μg of DNA per ml of cell suspension) and transferred to a 0.2 cm electrode gap chilled cuvette. A pulse of 2.5 kV/cm field strength, 6.8 ms time and 129 Ω set resistance was applied, cells were immediately suspended in 1.0 ml LB medium and incubated at 37°C for 1 h. Appropriate dilutions were plated on selective media.

### Plasmid methodology, enzymes and oligonucleotides

Plasmid and genomic DNA were purified in small scale according to Sambrook [50]. DNA fragments were purified from agarose gels with silica using GenElute™ gel extraction kit (Sigma). PCR-amplification of DNA fragments was carried out with Taq DNA polymerase (Promega). Cloning techniques were carried out by using standard methodologies [50]. Phage T4 polynucleotide kinase and T4 DNA ligase were from Amersham. Restriction endonucleases were purchased from Fermentas. Oligonucleotides were purchased from Sigma-Aldrich.

### Mapping of transposon insertion sites

Both *E. coli *K-12 strains MG1655 and DH5α were subjected to random mutagenesis with pLOFKm [25]. Six mini-Tn*10*::Km insertions in strains MG1655 and DH5α were sequenced to ascertain any site selection bias. All six insertions in DH5α were simple insertions. However, 4 of the MG1655 insertions contained integrated pLOFKm plasmid and only two were simple transposon insertions. It seems that, in addition to simple insertions, a *recA*^+ ^background allows the integration of plasmid pLOFKm DNA. For this reason DH5α was selected as the target for the construction of the Tn-insertion library.

The number of mutants estimated to cover the *E. coli *genome was calculated according to the Neutral-Base Pair Model described by Jacobs *et al. *[51]. In this model every base pair is assumed equally likely to define an insertion site (see below). Transposon Tn*10 *preferentially inserts at a particular 6 bp symmetrical consensus sequence (GCTNAGC; [52]). The consensus sequence inferred from the Tn*10 *insertions sequenced in this work indicated even a more relaxed specificity (GCNNNGC). There are 60,580 GCNNNGC sites in the MG1655 *E. coli *genome [53] equivalent to one insertion per 76 bp.

50 ng genomic DNA from each DH5α Km^r ^mutant was digested with *Csp6I *endonuclease. Five ng of the digested genomic DNA was religated in 20 μl final volume and incubated overnight at 16°C. Five μl of the ligation reaction were used as template for an inverse PCR reaction using oligonucleotides Tn10IR (CTGATGAATGTTCCGTTGCG) and Tn10Km (ACCTGGAATGCTGTTTTCCC). The amplified PCR-products were purified from agarose gels and both ends sequenced using Tn10IR and Tn10Km primers. DNA sequence homology search was performed with BLAST program from NCBI [54] to determine the position of the transposon insertion.

### Statistical analysis of the transposon insertions in DH5α

The coverage of the *E. coli *genome in the Tn-generated random mutant library was estimated according to a Neutral-Base Pair Model [51]. The number of times an ORF is hit follows a multinomial distribution with parameters *n, p*_1_,..., *p*_k_, where *n *is the number of transposon insertions assayed (20,000 in our case) *p*_j _is the probability of landing in the *j*th ORF, and *k *is the number of ORFs (4,390 for DH5α). *p*_j _was estimated as the length of the ORF divided by the total length of the *E. coli *genome (4,639,675 bp). All ORFs are included along with an extra "false ORF" that represents the entire noncoding region in *E. coli *(475,927 bp). When the model is applied to a set of 20,000 insertions, the expected number of missed ORFs is 394. Since 303 *E. coli *genes are essential for growth in rich media [23], more than 98% of the non-essential genes are expected to be hit at least once in a collection of 20,000 transposon insertions.

### Automated conjugation Assay

A whole-cell automated assay for conjugation, based on visible light emission [6], was carried out using a Biomek2000^® ^liquid handling robot (Beckman). A single colony of the donor strain was grown at 37°C in LB with Km and Ap overnight. Individual colonies of mutants were inoculated in 96 deep well plates (Axigen) and grown overnight at 37°C with agitation. 200 μl of the donor strain were added to the wells of the recipient plates, each containing 200 μl of an individual recipient mutant. A copy of each mutant was generated for storage before adding the donor strain. For the experiments under surface mating conditions, 8 μl of each resulting conjugation mixture were spotted into 96 well black microtiter plates (Thermo Electron Corporation) containing 300 μl LB agar. Mating plates were incubated at 37°C for 300 min and light emission detected in a microplate luminometer (Fluoroskan Ascent; Thermolab Systems) during the next 30 min (one measurement every 5 min) in order to corroborate that light production was increasing during this period of time. Light-x was the light produced by each colony at 330 min. Light-wt was defined as the median light value of the entire plate during the same period of time (excluding the negative controls). When potential mutants were collected on a single plate for a second assay, eight wt samples were included to calculate Light-wt (Average among them). Arbitrary Light Units (ALU) for a given mutant was defined as the decimal logarithm of the maximum value of light produced by this mutant (Light-x) divided by the light produced by the wild type (Light-wt), that is, ALU = log_10 _(Light-x/Light-wt).

### Standard conjugation experiments

Donor and recipient strains, grown to late exponential phase, were washed in LB and mixed in a 1:1 ratio. Mating mixtures were resuspended in 30 μl LB and deposited onto sterile nitrocellulose filters of 0.45 μm pore size. Filters were incubated for 1 h. at 37°C on the surface of LB-agar plates. Then, they were resuspended by vortexing and diluted in liquid medium. Transconjugants were selected on plates supplemented with appropriate antibiotics. The transfer frequency was expressed as the number of transconjugants per output recipient. Transfer rates were normalized to the wt strains (BW25113 or DH5α) and expressed as a percentage.

## Authors' contributions

DPM carried out all the experimental procedures of the study, participated in its design and drafted the manuscript. FC conceived the study, participated in its design and coordination and helped to draft the manuscript. Both authors read and approved the final manuscript.
